# Effect of Strontium Substituted Tetracalcium Phosphate Cement on Proliferation and Mineralization Potential in Human Dental Pulp Stem Cells

**DOI:** 10.14744/eej.2021.98704

**Published:** 2021-12-21

**Authors:** Nazwin BASHEER, Manavalan Madhana MADHUBALA, Jayasree R, Sekar MAHALAXMI, Sampath Kumar TS

**Affiliations:** 1. Department of Conservative Dentistry and Endodontics, SRM Dental College, Ramapuram, Chennai, India; 2. Department of Biomedical Engineering, G.K.M. College of Engineering and Technology, New Perungulathur, Chennai, India; 3. Department of Metallurgical and Materials Engineering, Indian Institute of Technology Madras, Medical Materials Laboratory, Chennai, India

**Keywords:** Dental pulp stem cell, mineralization, mineral trioxide aggregate, strontium, tetracalcium phosphate

## Abstract

**Objective:**

The aim of this study was to comparatively evaluate the proliferation, differentiation and mineralization inducing potential of strontium incorporated tetracalcium phosphate cement (STTCP) and mineral trioxide aggregate (MTA) on human dental pulp stem cells (hDPSCs).

**Methods:**

hDPSCs were cultured from freshly extracted human premolar teeth. Cement discs (5mm×3mm) were prepared using MTA and STTCP. hDPSCs were exposed to the test materials and grouped as follows: 1. MTA; 2. STTCP; 3. NC: Media alone (negative control; 4.PC: hDPSCs with osteogenic medium (positive control); Cell viability and proliferation was evaluated using MTT and trypan blue assays for 0, 7 and 14 day intervals. Odontoblastic differentiation potential were evaluated using ALP assay followed by degree of mineralization using Alizarin Red test and Osteopontin expression on day 7,14 and 21. Quantitative analysis were done by evaluating the absorbance and expressed as optical density. Statistical analysis was performed using Kruskal Wallis test followed by Friedman test (P<0.05).

**Results:**

MTA showed higher percentage of cell proliferation than STTCP at day 7 and 14. ALP assay revealed higher significant value for STTCP on day 7 and 14. STTCP exhibited intense staining and similar mineralization potential with MTA at all time periods. Osteopontin expression was exhibited by both MTA and STTCP on all days (P<0.05).

**Conclusion:**

STTCP promoted cell viability and enhanced mineralization and odontogenic differentiation potential on hDPSCs similar to MTA. STTCP has a potential to be an alternative therapeutic agent for pulp capping procedures.

## Introduction

The success of vital pulp therapy lies on the potential of the pulp to regenerate lost dentin. The amount, rate and quality of reparative dentin formation depend on the selection of appropriate bioactive pulp capping agent with good sealing ability and enhanced antibacterial activity (1). Numerous materials have been widely used for pulp capping procedure such as calcium hydroxide (CH), mineral trioxide aggregate (MTA) and biodentine (BD).

**Highlights**

• Strontium incorporated Tetracalciumphosphate (STTCP) is a bioactive and biocompatible cement.
• It has cell proliferation, differentiation and mineralization potential on hDPSCs similar to MTA thus serving as a promising alternative for vital pulp therapy.
• It can positively influence the healing of pulp which can result in reparative dentine formation.

Though CH was earlier considered as gold standard pulp capping material, it displays dentine bridge with tunnel defects which fail to provide hermetic seal against secondary infection (2). MTA is a highly successful bioactive material which also exhibits certain limitations such as longer setting time, difficult handling characteristics, discolouration potential (3). More recently, BD, a calcium silicate based bioceramic material, reported to have higher mechanical properties than MTA (4). Despite its superior properties, BD demonstrates poor radio-opacity and lower wash out resistance (5).

Moreover, all these materials have the ability to stimulate odontoblast to form reparative dentine on releasing high quantity of calcium and hydroxyl ions by creating alkaline environment (6). This high pH causes initial superficial necrosis that leads to temporary degeneration of underlying mesenchymal cells. In addition, prolonged contact of calcium silicate based cements also adversely affects the integrity of underlying dentin collagen matrix (7).

In recent years, calcium phosphate (CP) based materials have received a lot of attention due to their chemical similarity to bone and teeth (8). They are bioactive synthetic materials with excellent biocompatibility. Most of the calcium phosphate based cements have proven to have efficient dentinogenic potential as pulp capping material, Frequently, there are used in various forms such as hydroxyapatite (HA) and tricalcium phosphate (TCP-α, β), tetracalcium phosphate (Ca_4_(PO_4_)_2_O -TTCP), also known as hilgenstockite (9).

TTCP is one of the emerging cements with efficient bioactivity. It was first described by Hilgenstock in 1883. It was found to exist as tabular crystals as the basic component and phosphate-rich slag obtained during steel production which was extensively used as fertilizer in the past and as a ceramic biomaterial in phase-pure TTCP. TTCP in particulate form is an important ingredient in self-setting bone cements, which form HA in a continuous dissolution–precipitation process. It is the only phase with a calcium phosphate ratio greater than stoichiometric HA and has greater solubility in water (10). Studies have shown that TTCP has the ability to promote osteogenesis by increasing collagen synthesis and calciﬁcation of the extra-cellular matrix with no signiﬁcant inﬂuence on cell proliferation (11). Yoshimine & Maeda (11) showed that TTCP exhibited dentin bridge formation without necrosis or marked inflammation of the pulp. However, CH released from TTCP has limited antimicrobial activity due to the buffering action provided by dentinal tissues (12).

Strontium (Sr), as similar to calcium, is naturally present in the body and tooth structure in trace amounts. Portland cement and MTA have also been reported to contain heavy metals, such as manganese Sr, barium, uranium, cerium, mercury, cobalt, and silver (13). Addition of a small amount of Sr decreases the setting time of NanoMTA without any deleterious effect on its physical properties and with acceptable resistance to acidic environments by reducing its particle size (14). It has been proved that incorporation of a small amount of Sr to bone cements induces bioactivity and bio- conductivity properties (15). Sr incorporated Bioactive glass can stimulate dentinogenesis in human dental pulp stem cells (hDPSCs) by promoting their proliferation, differentiation and mineralisation in vitro likely to be beneficial in maintenance of vitality of tooth (16).

In order to enhance antibacterial activity of TTCP, a novel strontium substituted tetracalcium phosphate (STTCP) cement has been recently developed and characterized, which has the ability to release hydroxyl and strontium ions (17). Its application is being widely used in preventive dentistry because of its anticariogenic action and improved radiopacity due to its high atomic number (18). Pina et al. (19) developed a bone cement by modifying α-TCP with substituting calcium for strontium and found to have more amount of brushite formation in the novel cement with improved ion release, setting time and compressive strength. There were studies on strontium added to bioactive glass and fluoride, which showed improved antibacterial efficacy and remineralization potential (20). Jayasree et al. (17) showed that 10% STTCP (10SC) enhanced antibacterial activity and also remineralized the demineralized dentin superior to calcium hydroxide cement. Moreover, 10SC remineralized dentin exhibited the highest increase in hardness and elastic modulus. Sr has also been shown to stimulate dentinogenesis in human dental pulp stem cells (hDPSCs) by promoting their proliferation, differentiation and mineralization in vitro to produce tertiary dentine.

However, no literature reports are evidenced for the comparative evaluation of the cytocompatibility and odontogenic differentiation of STTCP on hDPSCs with MTA. Hence, the aim of this study was to comparatively evaluate the effect of the STTCP and MTA on proliferation, differentiation and mineralization inducing potential on hDPSCs. The proposed null hypothesis was that STTCP and MTA do not show any difference between them in odontogenic inducing potential on hDPSCs.

## Materials and Methods

The whole experimental protocol was performed following approval from Institutional Review Board (IRB no: SRMDC/ IRB/ 2017/ MDS/No.306) and after obtaining informed consent.

### Preparation of the experimental materials

STTCP powder and liquid components were prepared in the Department of Metallurgical and Materials Engineering, Indian Institute of Technology Madras, Chennai, while MTA was commercially procured (Angelus, Londrina, PR, Brazil). The composition of experimental materials is listed in Table 1. For fabrication of STTCP cement, the liquid and powder were dispensed on a glass slab at a ratio of 0.5mL/g and mixed with a stainless steel spatula to a paste like consistency, under aseptic conditions. Similarly, the experimental cement and MTA were sterilized with ethylene oxide and mixed to a consistency in a laminar flow hood MTA was mixed according to the manufacturer instructions. Following this, cement discs were prepared using respective sterile molds (diameter 5mm×height 3mm) and left to set for 4 hours at 37 °C in a humidified 5% CO_2_ incubator.

**Table 1.** Composition and source of experimental materials

	Materials	Composition	Source
1	MTA	Powder: SiO_2_, K_2_O, Al_2_O_3_, Na_2_O, Fe_2_O_3_, SO_3_, CaO, Bi_2_O_3_, MgO, KSO_4_, NaSO_4_, crystalline silica Liquid: Distilled water	MTA Angelus, Londrina, PR, Brazil
2	STTCP	Powder: 10 % strontium substituted tetra calcium phosphate. Liquid: 1M Na_2_HPO_4_, 10 wt % citric acid	Prepared in IIT Madras, Chennai, Tamil Nadu, India

MTA: Mineral trioxide aggregate, STTCP: Strontium Substituted Tetracalcium phosphate Cement, PR: Parana

### Grouping

Groups 1 and 2 consisted of the experimental materials MTA and STTCP respectively. Odontogenic medium containing 2 mL Dulbecco’s modified Eagle’s Medium (DMEM) (Gibco® Life Technologies™, NY, USA) supplemented with 10 mmol/L glycerol 2-phosphate, 0.2 mmol/L, L-ascorbic acid 2-phosphate, 0.1 mmol/L dexamethasone, and 0.1mmol/L 1, 25-dihydroxy vitamin D3 was used as positive control (group 3 - PC), while DMEM solution alone was used as negative control (group 4 - NC).

### Isolation, culture and seeding of hDPSCs

hDPSCs were obtained from caries free premolars that were freshly extracted for orthodontic purposes from healthy patients aged 16 to 26 years at SRM Dental College, Chennai, India. Teeth with caries, crack, resorption and fractures, and extracted for periodontal reasons were excluded. The teeth were washed several times in sterile phosphate buffer solution (PBS) (Merck KGaA, Darmstadt, Germany), then with normal saline (0.9% w/v sodium chloride, Merck KGaA, Darmstadt, Germany) followed by immersion in 1% povidone iodine for 2 min, immersion in 0.1% sodium thiosulfate for 1 min and again washed in sterile PBS. After which, a longitudinal furrow was made using a sterile flexible diamond disc without touching the pulp tissue. The teeth were vertically split with a dental surgical elevator and pulp tissue was gently separated with a sterile dentinal excavator from the crown and root and the pup tissue was carefully extracted. It was kept in incubator at 37°C for 40 minutes in 0.1% collagenase I (Gibco17100, Life Tech, NY, USA) to digest the tissue and liberate the cells. Following this, cell suspensions were seeded in tissue culture flasks (5×10^5^ cells/flask), consisting of MesenPRO RS™ basal medium supplemented with 20% MesenPRO RS™ growth supplement (Gibco® Life Technologies™, NY, USA), L-Glutamine with concentration of 2 mM, 100 U/ml penicillin, 100 μg/ml streptomycin and 25 μg/ml amphotericin B (Gibco® 15240, Life Technologies™, NY, USA). Culture of suspended cells was maintained at 37°C with 5% CO_2_ and 21% O_2_ in a humidified atmosphere. The medium was changed every 3 days, followed by subculturing through successive passaging at 1:3 ratios until cell seeding was done.

### Cell seeding onto the experimental materials:

The cultured hDPSCs at a density of 2×10^4^ cells/well, were seeded into 12-well plates containing the respective test materials (MTA, STTCP) and control groups. The cells thus seeded were used for evaluating the following parameters. All the experiments were performed in triplicate and average values were tabulated.

### Cell viability and proliferation assay

Following cell seeding, 200 μL MTT [3-(4,5-Dimethylthiazol-2-yl)-2,5-diphenyl tetrazolium bromide] solution was added to each well at three time intervals; immediately, after 7 and 14 days, and the cells were incubated for an additional 3 hours. The resulting MTT formazan crystals were dissolved by removing the culture medium and adding 200 μL dimethyl sulfoxide (Merck KGaA, Darmstadt, Germany) to each well. The plate was shaken at room temperature for 10 minutes to dissolve the crystals. Then, 100 μL of the solution in each well was transferred to a 96-well plate (C 980040, Tarsons, India) for absorbance determination at 570 nm with a microplate reader (Readwell Touch, India). The values were obtained as optical density (OD) and expressed as the mean±standard deviation.

### Cell count and morphological analysis

An equal volume of 200μl of 0.4% Trypan blue solution was added and gently mixed in a test tube and added to seeded cell suspensions at different time intervals of 0, 7 and 14 days. It was allowed to stand for 5 mins at room temperature to stain the cells. 10 μl of the stained cells were then placed in a haemocytometer and the number of viable (unstained) and dead (stained) cells were counted. Morphology of the cells was analysed under microscopic method. The percentage of viable cells was calculated as follows.



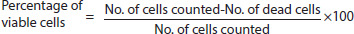



### Alkaline phosphatase assay:

hDPSCs samples collected at 7, 14 and 21 day intervals after cell seeding on the experimental materials were washed with PBS and then sonicated with a cell disruptor (Maxsell MS 300SH-9LQ, Tamil Nadu, India). Equal amount of cells (1x10^5^) samples were added into 96-well plates. ALP activity in the supernatant was determined using p-nitrophenyl phosphate ALP Assay Kit. The wells were brought up to an appropriate level with assay buffer, and 5 mM/L p-nitro phenyl phosphate was added. Following this, incubation was done for 60 mins at room temperature and reaction was stopped by adding 0.1 M NaOH. Absorbance value was measured as OD at 405 nm using microplate reader (Readwell Touch, India).

### Calcific nodule formation:

To visualise the presence of calcified nodules, cell cultures were rinsed with PBS, at 7, 14 and 21 day intervals and fixed in ice cold 50% ethanol for 10 minutes, rehydrated with 1 mL distilled water for 5 minutes, and then stained with 200 mL 1% alizarin red S for 3 mins at room temperature. Following the staining procedure, the cultures were washed 3 times with distilled water followed by 70% ethanol and viewed with imaging scans to assess the absorbance value for OD.

### Osteopontin Expression:

The cells were seeded with all experimental material in six well plate. The cells were incubated till the required time for 7, 14 and 21 days. After confluency the culture medium was aspirated and gently washed with PBS (Merck KGaA, Darmstadt, Germany) at room temperature. The wells were incubated with freshly prepared 4% paraformaldehyde - PBS at room temperature for 10 mins for fixation and washed in PBS for 2 minutes. The wells were incubated in 0.5% Triton X-100 in PBS at room temperature for 5 minutes for permeabilization. To reduce the background staining, the wells were blocked 5% Bovine serum albumin (BSA) prepared in PBS for 1 hour at room temperature. Indirect immunoperoxidase staining was performed with rabbit anti-mouse osteopontin polyclonal antibody as primary antibodies. Osteopontin primary antibody dilutions in 1% normal serum was prepared and incubated for overnight at 4°C. The wells were washed gently in PBS three times for 5 minutes each. Goat Anti Rabbit HRP conjugated secondary antibody (AB1870 Merck KGaA, Darmstadt, Germany) was prepared in 1% normal serum. The wells were incubated in the secondary antibody dilution for 1 hour at room temperature in a dark environment. The wells were washed gently in PBS for three times 5 minutes each and mounted to visualize immunoreactivity for osteopontin expression under light microscope (Olympus CX23 Biological Microscopes Mumbai,India).

### Statistical analysis

Statistical analysis was done using SPSS software version 22.0. Shapiro Wilk test was applied to find out the normality distribution. The data were evaluated by Kruskal Wallis test followed by Bonferroni posthoc test and Friedman test for intergroup and intragroup comparison for the interpretation of proliferation, mineralization and differentiation potential of the experimental materials. The significance level was set at P<0.05.

## Results

### hDPSCs Culturing, Cell viability and proliferation assay:

**Figure 1.**
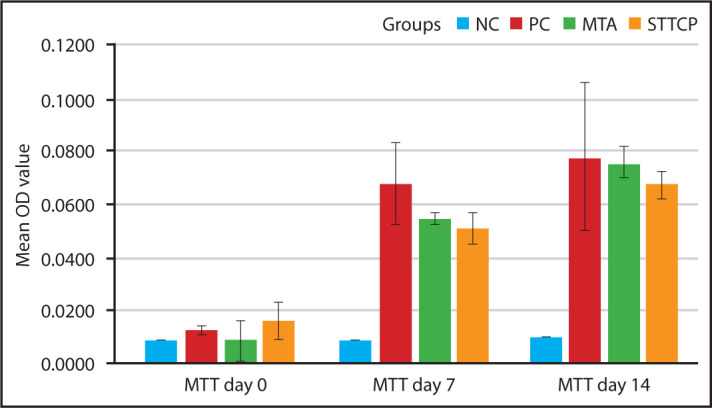
Clustered bar graph showing mean OD value for MTT assay of all the groups on day 0, 7 and 14 OD: Optical density, MTT: [3-(4,5-Dimethylthiazol-2-yl)-2,5-diphenyl tetrazolium bromide]

The cultured stem cells observed under light microscopy showed more round shaped morphology after 12 days following the third passage. MTA and STTCP groups maintained cell viability till 14^th^ day, without any significant difference between 7^th^ and 14^th^ day (Fig. 1). However, the percentage of cell proliferation was more in MTA for 7 and 14 days (84.5%, 97.1%) when compared to STTCP (73.9%, 81.9%).

### Cell count and morphological analysis:

**Figure 2.**
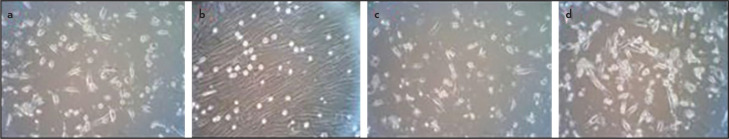
(a-d) Microscopic analysis of cell morphology under 200× magnification: MTA group on day 7 (a) and day 14 (b) STTCP group on day 7 (c) and day 14 (d) MTA: Mineral trioxide aggregate, STTCP: Strontium Substituted Tetracalcium phosphate Cement

STTCP and MTA did not show statistical difference in cell count at all time periods (P>0.05). The percentage of viable cells was also in accordance with the cell count as seen in Table 2. The microscopic analysis exhibited increased number of fibroblast like cells with spindle shaped morphology and round undifferentiated stem cells on day 7 in both the groups. STTCP showed pronounced cell differentiation than MTA which can be evidenced by the presence of more number of polygonal cells with branching in multiple layers in an intertwined fashion (Fig. 2 a-d).

**Table 2.** Percentage of cell viability derived using Trypan blue assay of all the groups on day 0, 7 and 14

Groups	Day 0	Day 7	Day 14
NC	0.00±0.00	0.00±0.00	0.00±0.00
PC	99.23±0.15	96.71±0.24	98.47±0.062
MTA	99.80±0.79	97.33±0.22	98.62±0.39
STTCP	99.60±0.14	97.78±0.37	99.21±0.19

NC: Negative control, PC: Positive control, MTA: Mineral trioxide aggregate, STTCP: Strontium Substituted, Tetracalcium phosphate Cement

### Alkaline phosphatase assay:

**Figure 3.**
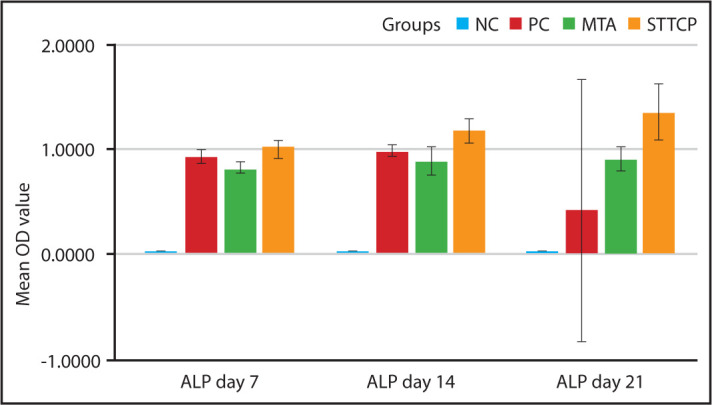
Clustered bar graph showing mean OD value for ALP assay of all the groups on day 7, 14 and 21 OD: Optical density, ALP: Alkaline Phosphatase asssay

STTCP showed statistically greater ALP activity than MTA on day 7 and 14, whereas there was no difference between them on day 21 as shown in Figure 3. PC values were between MTA and STTCP, with no significant difference between them. On day 21, PC showed a major decline in ALP activity when compared with experimental groups. As shown in Figure 3, STTCP showed gradual increase in ALP activity from day 7 to 21 without any significant difference.

### Calcific nodule formation:

**Figure 4.**
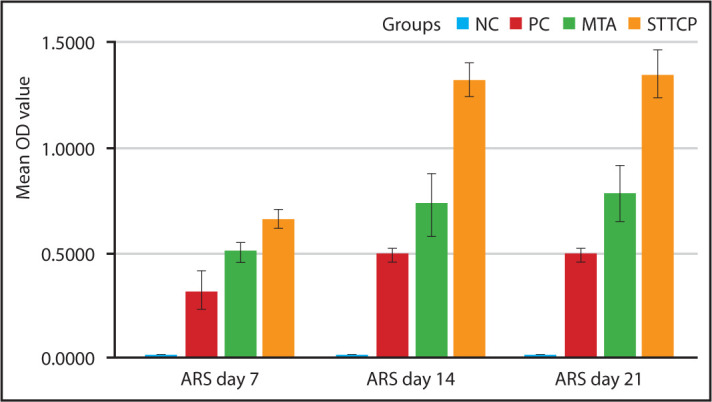
Clustered bar graph showing mean OD value for ARS assay of all the groups on day 7, 14 and 21 OD: Optical density, ARS: Alizarin Red staining

**Figure 5.**
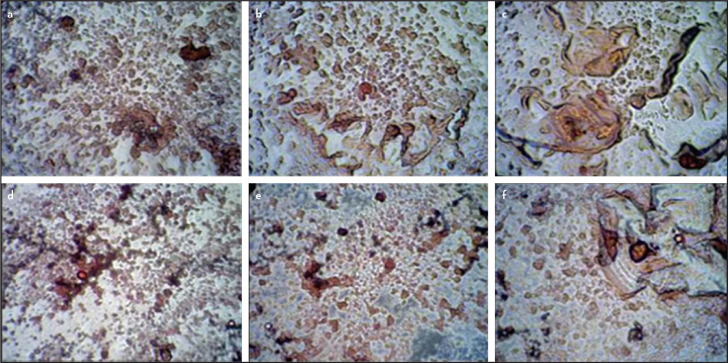
(a-f) Microscopic analysis of calcific nodule formation after ARS staining under 200× magnification: Calcific nodules can be visualized in MTA group on day 7 (a), day 14 (b), day 21 (c) ; in STTCP group on day 7 (d), day 14 (e) and on day 21 (f) MTA: Mineral trioxide aggregate, STTCP: Strontium Substituted, Tetracalcium phosphate Cement, ARS: Alizarin Red staining

The quantification of ARS confirms similar mineralization potential by STTCP and MTA at all time periods as shown in Figure 4. Initiation of calcific nodules were seen on day 7 in both the groups with gradual increase in mineralization potential from day 7 to 21. More intense staining was observed in STTCP group on day 21. The mineralization potential was confirmed with red calcific nodules formation which were evidenced by microscopic images (Fig. 5 a-f).

### Osteopontin immunoreactivity:

**Figure 6.**
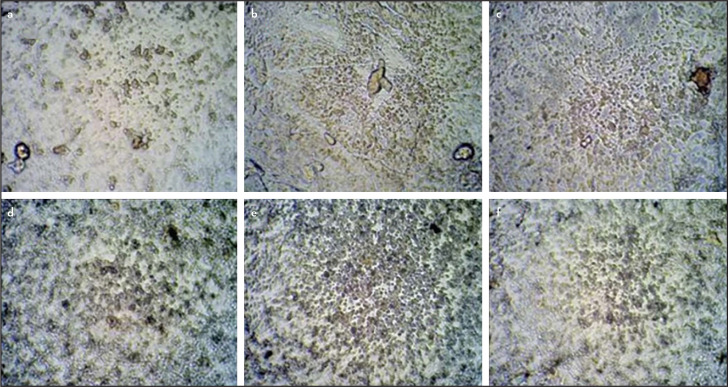
(a-f) Microscopic analysis of OST expression by indirect immunohistochemistry under 200× magnification: OST staining can be visualized in MTA group day 7 (a), day 14 (b) and day 21 (d); in STTCP group on day 7 (d), day 14 (e) and on day 21 (f) OST: Osteopontin, MTA: Mineral trioxide aggregate, STTCP: Strontium Substituted, Tetracalcium phosphate Cement

Osteopontin expression was exhibited by both STTCP and MTA on all days as evidenced by microscopic images (Fig. 6 a-f).

## Discussion

TTCP is commonly used for bone reconstruction due to its osteoconductivity and its similar crystallographic structure to bone (19). It reacts with other calcium phosphates with lower Ca/P ratio to form HA without producing any acidic or basic by products. Sr is known to be more reactive than Ca. Sr^2+^ ion substituted TTCP forms HA on hydrolysis, replaces calcium ion in the crystal structure thus enhancing the HA formation and antibacterial efficacy. There is high and sustained release of OH^−^ and increased release of Sr^2+^ ion at 10% concentration of Sr. It can also increase the radiopacity of the material (20).

The culture of hDPSCs was performed using standardized methodology as proposed by Perry et al. (21). According to MTT assay, STTCP showed similar cell viability as MTA at 7 and 14 days, that was significantly lesser than PC. This could be attributed to the presence of ions like Ca^2+^, PO_4_^3-^ and OH^−^and their influence on pH changes during setting. Ehara et al. (22) reported significant difference in the cell proliferation on MC3T3-E1 cells by TTCP for day 7 when compared to osteogenic medium as seen in this study. Ogata et al. (23) compared the cell viability of calcium phosphate cement with MTA and found to have no significant difference between days 3, 5 and 7. The results of our study rejected the proposed null hypothesis.

As MTT assay was measured over a time period, the increasing value of absorbance along day intervals 0, 7 and 14 can be interpreted as an indirect measurement for cell proliferation. The percentage of cell proliferation was found to be higher in MTA than in STTCP on 7 and 14 day interval. This indicates that the number of viable cells and the proliferation rate has increased with time in MTA than STTCP. This could be due to the presence of 10% Sr in STTCP. Huang et al. (18) showed higher concentration (10 mM) of Sr tend to inhibit the proliferation of hDPSCs. Sr enhances the cell viability in lower concentrations (25-500 μM) while higher concentration above 1000 μM has been reported to have greater rate of cell apoptosis. This effect is due to the activation of extracellular signal-related kinase (ERK) signalling pathway and B-Cell lymphoma family mediated apoptosis (24). In previous study, 8% Sr substituted TTCP loaded with ornidazole showed higher cell proliferation rate during first 3 days (25). Hence, in our study, the higher amount of Sr can be a reason for the slight decrease in cell proliferation of hDPSCs as seen on day 7 and day 14. However, Bizelli- Silveira et al. (26) reported that an increased concentration of Sr could enhance cell viability with time on human periodontal stem cells.

In trypan blue assay, similar results were observed for both STTCP and MTA in terms of cell counting and percentage of viable cells as given in Table 2. On morphological analysis at day 7, STTCP and MTA showed predominantly spherical cells, which denotes undifferentiated pulp stem cells, with colony forming unit cells and few spindle shaped cells (Fig. 2a, c). At day 14, there was more number of spindle shaped cells in both the groups depicting the differentiated cells (Fig. 2b, d). However, the branching of the spindle-shaped cells with multiple layer growing pattern in an intertwined fashion were more in STTCP compared to MTA. This could be attributed to the fact that the initiation of cell differentiation by STTCP would be earlier for STTCP than MTA. On contrast, Lee et al. (27) showed that MTA had better proliferation rate than calcium phosphate cement at day 14.

Generally, as the proliferation rate decreases, the mineralization phase begins. The first mineralization marker which can be detected is alkaline phosphatase enzyme as it releases from the matrix bound vesicles, its increase reflects a shift in the differentiation of hDPSCs cells. Calcium deposits are also an indicator of successful differentiation of stem cells into odontoblasts and in vitro dentin-formation. STTCP showed a significant increase in ALP activity than MTA on days 7 and 14 alone. Calcium deposits can specifically be stained bright orange-red using Alizarin Red (28). However, quantitative assessment of ARS showed no significant difference between STTCP and MTA groups during all time periods. Microscopic evaluation of ARS showed increased presence of calcific nodules. STTCP group also showed intense expression of osteopontin (Fig. 6 a-f) as evidenced by both the microscopic images. Previous studies on TTCP showed an enhanced ALP activity on clonal pre-osteoblastic cells with increased mineralized nodule formation (18, 27, 28). The mineralization potential of STTCP could be attributed to its accelerating effect on osteoblastic differentiation in early phase, promoting matrix production. Calcium is a modulator of intracellular events of mineralization. The stem cells possess voltage activated calcium channels in plasma membrane. As TTCP increases the concentration of Ca ions, there could be an enhanced entry of these ions intracellularly. An intensified mineralization can be observed at the genetic level as it supplies calcium and phosphate.

Further, interaction of Sr in STTCP could stimulate odontoblastic cells in all phases of dentin formation, therefore leading to superior mineralization. Previous studies with Sr on hDPSCS showed significant increase in odontogenic potential than Sr free groups. Similar finding was reported by Aimaiti et al. (24) where Sr showed significant increase in calcium nodule formation on 14 and 21 day intervals on human adipose stem cells. Sr could activate calcium sensing receptors (18) in the cells leading to further activation of mitogen activated protein kinase (MAPK) signalling pathway which is responsible for cellular events of growth and differentiation (29). Sr also acts on early osteoblastic precursors to induce COX-2 (30) and increase PGE2 production, thus, enhancing the osteoblastic differentiation and mineralization (31). Further, Sr might also interact with fibroblast growth factor receptors present in the hDPSCs thus promoting odontoblast synthetic activity (32).

STTCP promotes cell viability and enhances odontogenic mineralization potential on hDPSCs similar to MTA so it could be an alternative therapeutic agent for pulp capping. It can positively influence healing of pulp followed by odontogenesis resulting in reparative dentin formation. Further, animal and clinical studies have to be explored to introduce STTCP into clinical practice.

## Conclusion

Within the limitations of this study, it can be concluded that STTCP is bioactive and biocompatible material capable of cell proliferation, mineralization and odontoblastic differentiation on hDPSCs similar to MTA.

### Disclosures

**Acknowledgement:** We are pleased to acknowledge Dr.Naresimhan Srinivasan MDS., who contributed to the statistical part of the manuscript preparation.

**Conflict of interest:** The authors deny any conflict of interest.

**Ethics Committee Approval:** This study was approved by the Ethics Committee of The Institutional Review Board SRM Dental College (Date: 05/10/2019, Number: SRMDC/IRB/2017/MDS/No.306).

**Peer-review:** Externally peer-reviewed.

**Financial Disclosure:** This study did not receive any financial support.

**Authorship contributions:** Concept – N.B., M.M.M., J.R., S.M., S.K.T.S.; Design – N.B., M.M.M., S.M.; Supervision – N.B., M.M.M., S.M.; Funding - N.B.; Materials - N.B., J.R., S.K.T.S.; Data collection &/or processing – N.B., M.M.M., S.M.; Analysis and/or interpretation – N.B., M.M.M., S.M.; Literature search – N.B., M.M.M., S.M., J.R., S.K.T.S.; Writing – N.B., M.M.M., S.M.; Critical Review – J.R., S.K.T.S., M.M.M., S.M.
